# P-1586. Safety and Efficacy of Vitamin D3 in the Management of COVID-19: A Systematic Review and Meta-Analysis

**DOI:** 10.1093/ofid/ofaf695.1765

**Published:** 2026-01-11

**Authors:** Ahmed Hosney Nada, Ismail A Ibrahim, Nada Khalid Asar, Mohamed Wagdy, Heidi Sherif Farouk, Masah Ateeq, Mariam Mahmoud Mohammed, Abdulrahman Qenawy

**Affiliations:** Faculty of Medicine, Benha University, Egypt, Quesna, Al Minufiyah, Egypt; Fenerbahce University, Faculty of Health Sciences, Istanbul, Istanbul, Turkey; Mansoura university, Giza, Al Jizah, Egypt; modern university for technology and information,Cairo,Egypt, basil, Kafr ash Shaykh, Egypt; Faculty Of medicine, Alexanderia University, Egypt, Alexanderia, Al Iskandariyah, Egypt; Faculty Of Medicine, Port-Said University, Egypty, Jenin Camp, Not Applicable, Palestinian Territories; Faculty of Pharmacy, Fayoum University, Fayoum, Egypt, Al fayyum, Al Fayyum, Egypt; Faculty of Medicine, South Valley University, Qena, Egypt, Qina, Qina, Egypt

## Abstract

**Background:**

The COVID-19 pandemic has had a profound global impact, resulting in millions of deaths and major disruptions to daily life. This has prompted the investigation of various therapeutic agents, including vitamin D3, due to its potential immunomodulatory effects. This systematic review and meta-analysis assessed the efficacy and safety of vitamin D3 in the management of COVID-19.

**Figure:**
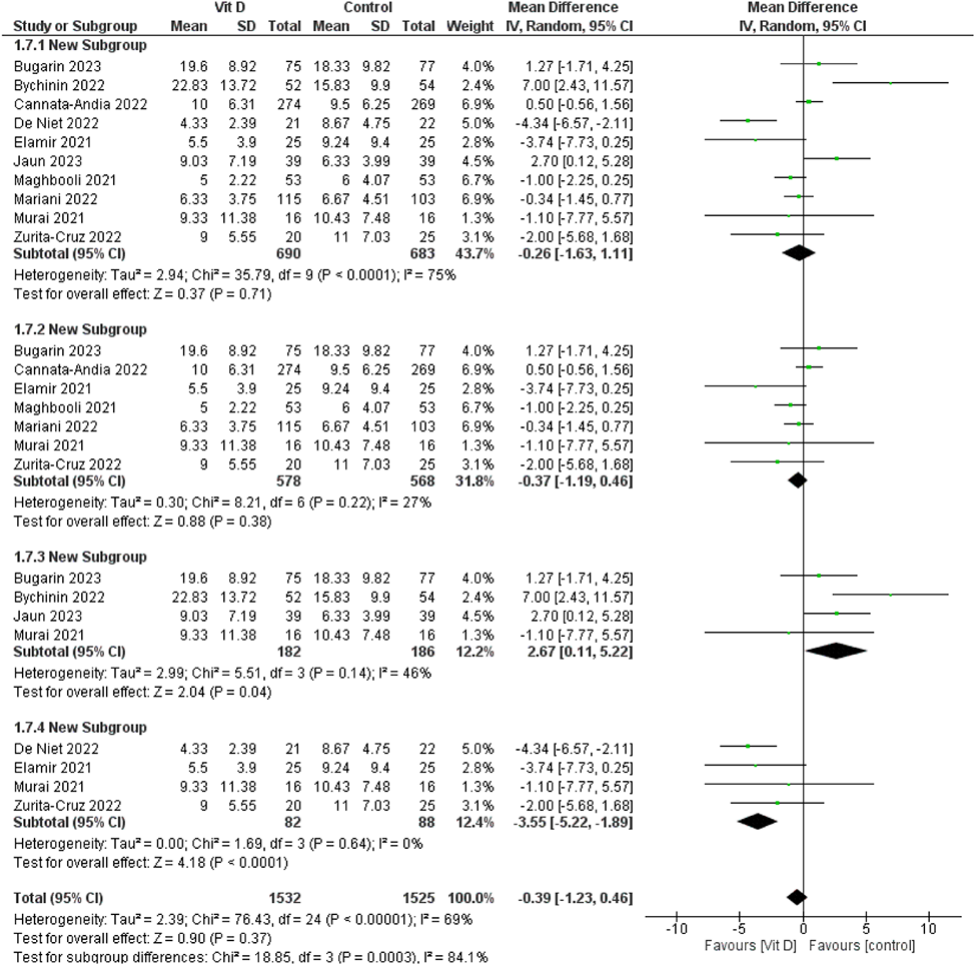

**Figure:**
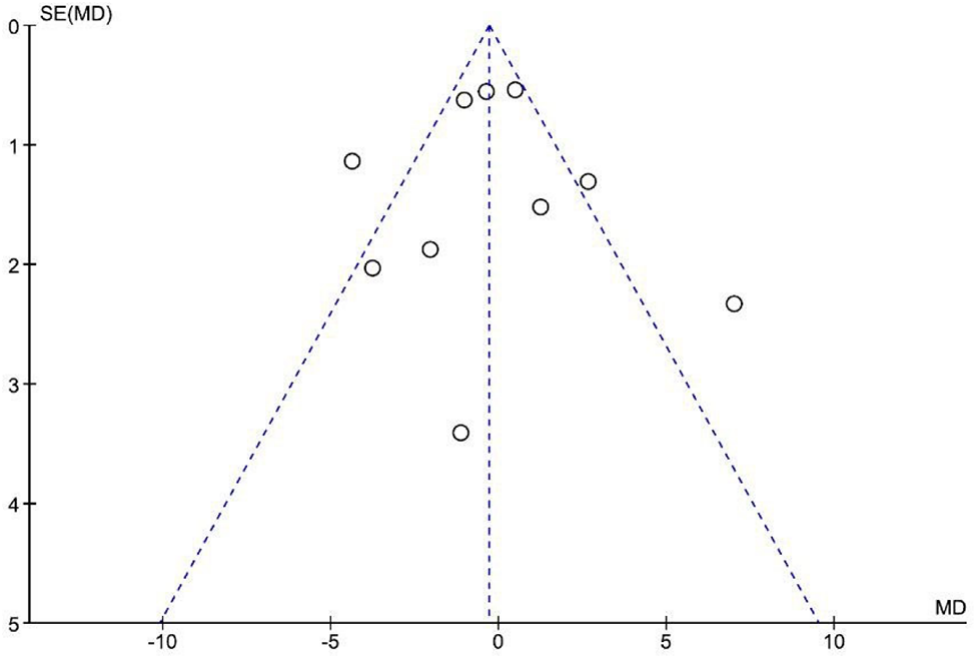

**Methods:**

A comprehensive literature search was conducted in PubMed, Scopus, Web of Science, and Embase up to March 2025. Primary outcomes included length of hospital stay, ICU admission, ICU length of stay, need for oxygen supplementation, and need for mechanical ventilation. Secondary outcomes included inflammatory markers ( C-Reactive Protein(CRP), D-dimer,Lactate dehydrogenase (LDH), and ferritin).

**Figure:**
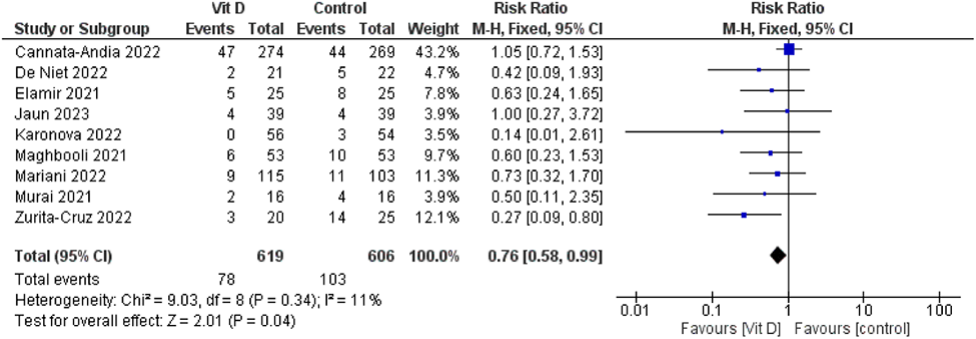

**Figure:**
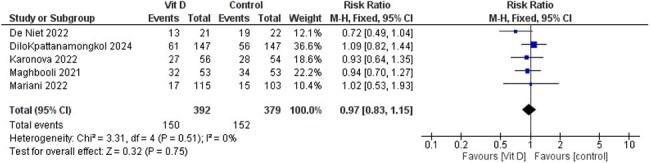

**Results:**

Seventeen randomized controlled trials with a total of 2,762 patients were included. Vitamin D3 was not associated with a significant reduction in hospital stay (MD = -0.26, 95% CI [-1.63 to 1.11], *P* = 0.71), oxygen supplementation (RR = 0.97, 95% CI [0.83 to 1.15], *P* = 0.75), or mechanical ventilation (RR = 0.88, 95% CI [0.66 to 1.17], *P* = 0.38). However, a significant reduction in ICU admissions was observed in the vitamin D3 group (RR = 0.76, 95% CI [0.58 to 0.99], *P* = 0.04). There was no significant difference in ICU length of stay (MD = 0.45, 95% CI [-1.78 to 2.67], *P* = 0.69). No significant differences were found in inflammatory biomarkers between the groups.

**Conclusion:**

Vitamin D3 supplementation may reduce ICU admissions in patients with COVID-19, though no significant effects were observed for other clinical or laboratory outcomes. Subgroup analyses suggest a potential reduction in hospital stay, warranting further high-quality research.

**Disclosures:**

All Authors: No reported disclosures

